# Aspen Wood Characteristics Following Thermal Modification in Closed Process Under Pressure in Nitrogen

**DOI:** 10.3390/ma17235930

**Published:** 2024-12-04

**Authors:** Guntis Sosins, Juris Grinins, Prans Brazdausks, Janis Zicans

**Affiliations:** 1 Latvian State Institute of Wood Chemistry, 27 Dzerbenes Str., LV-1006 Riga, Latvia; guntis.sosins@kki.lv (G.S.); prans.brazdausks@kki.lv (P.B.); 2 Faculty of Materials Science and Applied Chemistry, Institute of Polymer Materials, Riga Technical University, 3/7 Paula Valdena Street, LV-1048 Riga, Latvia; janis.zicans@rtu.lv

**Keywords:** aspen, thermal modification, nitrogen, pressure, moisture, strength

## Abstract

Using a pilot-scale chamber with an interior capacity of 340 L, European aspen (*Populus tremula*) wood boards were thermally modified (TM) under pressure in nitrogen at a maximum temperature of 160–170 °C, for 60–180 min, and with an initial nitrogen pressure of 4–5 bar. After the TM process, aspen wood was characterised by dimensional changes, mass loss (ML), equilibrium moisture content (EMC), antiswelling efficiency (ASE), cell wall total water capacity (CWTWC), modulus of rupture (MOR), modulus of elasticity (MOE), and Brinell hardness (BH). This work offers fresh insights into the characteristics of aspen wood following a closed TM process in pressurised nitrogen. TM caused ML of 5.4–14.5% and shrinkage in all anatomic directions. The ASE ranged from 22 to 70%, while the CWTWC was reduced from 35% to 11–27%. After treatment, EMC and volumetric swelling (VS) were more than twice as low as in untreated wood. Although MOE values increased and the average MOR reduced following TM, the changes were not important. The TM aspen wood tangential surface’s BH dropped and was noticeably lower than the radial surface’s BH.

## 1. Introduction

European aspen (*Populus tremula*) wood is light and soft, with low bending strength and stiffness and medium shock resistance. It is utilised in the paper and pulp industries, as well as the match industry. Chipboards for carpentry, roofing chips, barrel boards, and sauna room interior décor are all made from aspen wood. Aspen wood has a poor heat capacity; yet, burning it removes soot from chimneys. As with most wood species, aspen wood has many drawbacks, such as high moisture and water absorption, low biological durability, soft surface, poor weathering, and dimensional stability.

Wood preservatives are frequently employed, allowing wood to be used in severe settings, including tropical climates and coastal pilings. Preservative treatments, despite their ability to protect wood from decay and insects, have little effect on wood qualities such as dimensional instability. Wood preservatives are registered pesticides, and their continued significance is dependent on development within the constraints of use imposed by environmental concerns. Most treated wood must be disposed of in a landfill or burnt in specialised incinerator facilities. Unlike wood preservatives, wood modifications change the polymeric components of wood to attain a desired feature. Modified wood is commonly defined as any wood that has received a chemical, physical, or thermal treatment to improve its qualities. Changes can improve dimensional stability while lowering hydrophilicity, even though many wood modification techniques aim to increase decay resistance.

Thermal modification (TM) is the most popular method for enhancing the characteristics of wood. In Europe, there are numerous wood TM processes with different pressure and oxygen exclusion levels. The most often used commercialised methods are Thermowood, Plato, Wood treatment technology, Feuchte–Wärme–Druck, Le bois Perdure, Rétification, Opel-therm-Vacuum, Termovuoto, Firmolin, Oil-heat treatment [1,2,3]. The specific conditions, descriptions of these procedures, and available production levels were all covered in our previous studies [4,5]. Nonetheless, a wide variety of TM wood products are now offered on the market as a result of the several manufacturers. The properties of the TM wood differ substantially based on the raw materials utilised or the process methods and conditions applied, even if all TM procedures adhere to the same fundamental idea. Additionally, the processes’ environmental loads, such as their energy consumption, emissions, and waste vary. Even if TM procedures are thought to be environmentally beneficial, there is frequently a lack of data to back this up [1,3,6].

The qualities of modified wood are influenced by TM parameters such as atmosphere, temperature, treatment duration, pressure, rate of heating and cooling, moisture content of the wood, and dimensions. Depending on whether a steam flow is used or a closed steam environment is used for the treatment, the impact of water vapour on wood has been extensively studied. While the impact of pressure on material properties has not been extensively studied in the literature, the time and temperature of the TM process are thought to be the most crucial variables. Choosing the proper TM settings allows you to achieve a perfect balance that improves dimensional stability, moisture resistance, and biological durability without drastically compromising the material’s mechanical strength. During modification, a lot of energy is needed to produce steam and collect the condensate. Because it is a readily available, inexpensive gas with a greater thermal conductivity coefficient—which means it takes less energy to heat—nitrogen is a more efficient environment. The amount of condensates produced by TM in nitrogen is quite small [7].

White birch (*Betula papyrifera*) and quaking aspen (*Populus tremuloides*) were subjected to a one-hour TM in humid inert gas at a temperature of 205–210 °C. In an accelerated ageing test conducted in a lab setting, specimens were coated with acrylic polyurethane containing organic UV stabilisers and acrylic polyurethane containing bark extract and lignin stabiliser. They were then subjected to UV radiation and water spray. When it came to protecting TM wood surfaces, acrylic polyurethane with lignin stabiliser and bark extracts worked better than organic UV stabilisers. Additionally, the addition of lignin stabiliser and bark extract enhanced the coating’s qualities because there were no surface cracks or checks visible [8].

White birch (*Betula papyrifera*) and quaking aspen *(Populus tremuloides*) were TM in a gas containing 80% nitrogen and 20% carbon dioxide, with water vapour added at 120, 160, 200, 220, and 230 °C. Birch’s chemical structure was more altered than aspen’s. The MOE of birch dropped, whereas the MOE of aspen increased with maximum TM temperature. Birch’s MOE, MOR, and hardness reduced after 200 °C. However, the MOE of aspen rose with the maximum TM temperature. Aspen’s hardness was substantially lower than that of birch. It showed a minor decrease in the radial direction, while in the tangential direction, the hardness values reached a maximum of approximately 160 °C. The considerable increase in BH of the birch and the slight and transitory increase in the MOR of both species at temperatures 160–220 °C is most likely attributable to lignin ramification [9]. Beech (*Fagus sylvatica*) wood was placed in an oven between metallic plates and heated to 230 °C under nitrogen or vacuum (200 mbar). For all specimens, the decrease in mechanical characteristics (MOE, MOR, BH) was lower in vacuum TM samples compared to nitrogen [10].

European (*Populus tremula* L.) and hybrid (*P. tremula × tremuloides*) aspen wood were dried using warm air drying, press drying, and TM at 180 °C using Thermowood technique. The radial Brinell hardness of press-dried specimens exceeded that of normally dried or TM specimens. MOE and MOR were at their peak in press-dried specimens. Regardless of drying technique, the tangential shear strength of European aspen specimens was approximately 5% greater than that of hybrid aspen. TM wood had considerably lower tangential shear strength values than conventionally dried wood. In both aspen species, TM specimens had considerably lower longitudinal tensile strengths than conventionally and press dried specimens [11]. Wood veneers of aspen (*Populus tremula* L.) and poplar (*Populus × canadensis Moench*) were TM by two processes–in steam under pressure at 160 °C for 50 min; by a 250 mbar vacuum process at 204–214 °C for 120 min; and 217–218 °C for 30–180 min. The lightness parameter L* dropped after TM, and the veneers were much darker. TM veneers were considered for decorative use as exterior lamination layers [12].

Our study’s primary objective was to ascertain the relationships between the water-related and mechanical characteristics of European aspen (*Populus tremula*) wood and the TM conditions in a nitrogen atmosphere. Compared to hardwood species like beech, birch, and poplar, or less common species like oak or ash, aspen wood’s TM has gotten much less attention. Data on the characteristics of aspen wood following TM in a nitrogen environment, either in an open or closed process or at ambient or increased pressure, is lacking from literature studies.

## 2. Materials and Methods

### 2.1. Materials

European aspen (*Populus tremula*) wood boards were purchased from local wood manufacturing company Apsītes AG (Vecpiebalga, Latvia) harvested in central and eastern Latvia. Eighteen European aspen wood boards, each 1000 × 100 × 27 mm^3^ (longitudinal × tangential × radial), were used by each TM. There were no obvious material flaws in the high-quality boards that were employed, such as knots, grain slop, bark, wanes, blue stains, decays, bug holes, rattles, or distortions. Prior to TM, all boards were conditioned in a normal climate (20 ± 2 °C, 65 ± 5% relative humidity). Randomly selected boards with an average density of 498 ± 30 kg × m^−3^ were utilised for TM. ISO 13061-1:2014 [13] was used to assess the moisture content of the wood, and ISO 13061-2:2014 [14] was used to determine its density.

### 2.2. Thermal Modification Procedure

TM was conducted in a 340 L pilot-scale chamber constructed by Wood Treatment Technology (Grinsted, Denmark) composed of stainless steel. The jacket was kept at a constant temperature by circulating hot mineral oil. Before being heated, the samples were autoclaved for 30 min at 0.2 bar of vacuum to remove oxygen. In order to provide the required starting pressure (4 or 5 bar), nitrogen was injected into the autoclave from a nitrogen gas cylinder after the vacuum process. In order to create a steam that would catalyse the hydrolysis of hemicelluloses, 1–1.5 L of tap water was first placed in the autoclave. The TM system remained sealed and static (no mixing took place) until the pressure release step. The TM characteristics for European aspen wood are shown in Table 1.

Process parameters were chosen based on earlier TM experiments in nitrogen with birch and pine wood [4,5]. After TM, boards with obvious flaws (cracks, bloating, twisting, etc.) were registered to describe the overall quality of the boards following processing. The entire TM process took 16–18 h at 160 °C and 18–19 h at 170 °C, including heating, holding temperature, and cooling.

### 2.3. Physical Parameters

Equation (1) was used to calculate the mass loss (ML) following each TM as a percentage of the initial mass of the completely dry (103 ± 2 °C for 24 h) wood:

(1) $$ML(\%)=\frac{\left(m_{0}-m_{TM}\right)}{m_{0}}\times 100$$

where:

- ML is the mass loss after TM in nitrogen [%];
- $m_{0}$ is the oven-dried mass of the boards before treatment [g];
- $m_{TM}$ is the oven-dried mass of the boards after TM in nitrogen [g].

Equation (2) was used to compute the volumetric changes after each treatment:

(2) $$V_{C}(\%)=\frac{\left(V_{U}-V_{TM}\right)}{V_{U}}\times 100$$

where:

- $V_{C}$ is wood volume changes after TM [%];
- $V_{U}$ is the of conditioned wood board volume before TM [cm^3^];
- $V_{TM}$ is the of conditioned wood board volume after TM [cm^3^].

Equation (3) was used to determine the wood board’s $V_{U}$ volume prior to TM:

(3) $$V_{U}=L_{U}\times {Tg}_{U}\times R_{U}$$

where:

- $L_{U}$ is the length of the untreated wood’s longitudinal direction [cm];
- ${Tg}_{U}$ is the length of untreated wood tangential direction [cm];
- $R_{U}$ is the length of untreated wood radial direction [cm].

Using Equation (4), the volume of wood board $V_{TM}$ following TM was determined:

(4) $$V_{TM}=L_{TM}\times {Tg}_{TM}\times R_{TM}$$

where:

- $L_{TM}$ is the length of the TM wood longitudinal direction [cm];
- ${Tg}_{TM}$ is the length of the TM wood tangential direction [cm];
- $R_{TM}$ is the length of the TM wood radial direction [cm].

### 2.4. Dimensional Stability

Under cyclic circumstances, the antiswelling efficiency (ASE) of fifteen specimens measuring 20 × 20 × 20 mm^3^ (L × T × R) was investigated. Specimens were conditioned in a typical climate (20 ± 2 °C, 65 ± 5% relative humidity) before each cycle. Five cycles of oven drying and full-cell saturation were carried out. The volumetric swelling (VS) coefficients of the treated specimens (S_t_) relative to the untreated controls (S_u_) were used to compute the ASE. Vacuum water impregnation (0.2 bar, 120 min) and water storage at 22 °C for 24 h comprised the soaking phase. During the drying process, there were seventy-two hours at 20 °C, eight hours at 45 °C, eight hours at 60 °C, eight hours at 80 °C, and eight hours at 103 °C. Average measurements of axial, tangential, and radial swelling served as the basis for the ASE estimation. Equation (5) was used to determine the ASE for each treatment:

(5) $$ASE(\%)=\frac{\left(S_{u}-S_{t}\right)}{S_{u}}\times 100$$

where:

- ASE is the antiswelling efficiency [%];
- $S_{u}$ is the untreated specimens’ volumetric swelling coefficient;
- $S_{t}$ is the TM specimens’ volumetric swelling coefficient.

Equation (6) was used to determine the volumetric swelling coefficients for each treatment:

(6) $$S(\%)=\frac{\left(V_{1}-V_{0}\right)}{V_{0}}\times 100$$

where:

- S is the volumetric swelling coefficient [%];
- $V_{1}$ is the volume of wood following a water soak [cm^3^];
- $V_{0}$ is the volume of oven-dried wood prior to water soaking [cm^3^].

Equation (7) was used to calculate the variations in cell wall total water capacity for each treatment:

(7) $$CWTWC(\%)=\frac{\left(V_{u1}-V_{u0}\right)}{m_{u0}}\times 100$$

where:

- CWTWC is the cell wall total water capacity [%];
- $V_{u1}$ is the volume of wood following a water soak [cm^3^];
- $V_{u0}$ is the volume of oven-dried wood [cm^3^];
- $m_{u0}$ is the specimens’ oven-dried mass before each soaking session [g].

### 2.5. Moisture Uptake

Fifteen TM wood specimens (20 × 20 × 20 mm^3^) were examined for moisture uptake from a constant humidity environment at relative humidity (RH) of 65, 75, and 98% at 20 ± 2 °C. The wood specimens were put in a conditioning room with a relative humidity of 65% when they were totally dry. Specimens were placed over saturated NaCl salt solution (RH of 75%) and CuSO_4_ × 5H_2_O salt solution (RH of 98%) in desiccators to condition the wood at higher RH levels. The specimens were kept at increasing relative humidity levels of 65, 75, and 98%. When the mass of the wood samples remained constant across three weightings at 48 h intervals (up to 30 days), dimensional changes and the equilibrium moisture content (EMC) were assessed. Equation (8) was used to calculate the EMC for each treatment:

(8) $$EMC(\%)=\frac{\left(m_{1}-m_{0}\right)}{m_{0}}\times 100$$

where:

- EMC is the equilibrium moisture content [%];
- $m_{0}$ is the oven-dry mass of the specimen;
- $m_{1}$ is the specimen’s equilibrium mass following conditioning at a specific relative humidity.

Equation (9) was used to determine the volumetric swelling (VS) of conditioned specimens at a specific relative humidity for each treatment:

(9) $$VS(\%)=\frac{\left(V_{1}-V_{0}\right)}{V_{0}}\times 100$$

where:

- VS is the volumetric swelling [%];
- $V_{1}$ is the equilibrium volume of the specimen after conditioning at a given RH [cm^3^];
- $V_{0}$ is the specimens’ oven-dried volume prior to conditioning [cm^3^].

$V_{1}$ and $V_{0}$ were calculated similarly to how they were in Equations (3) and (4).

### 2.6. Mechanical Strength Tests

The specimens were conditioned in a normal climate (20 ± 2 °C, 65 ± 5% relative humidity) before the mechanical strength tests. The modulus of rupture (MOR) and modulus of elasticity (MOE) tests were performed according to ISO 13061-3:2014 [15] and ISO 13061-4:2014 [16]. Using Zwick Roell Z010 material testing equipment (Ulm, Germany), the static flexural strength (3-point bending test) of 30 specimens measuring 360 × 20 × 20 mm^3^ was ascertained. Using the Zwick Roell Z100 material tester (Ulm, Germany), the Brinell hardness of ten specimens per treatment, each measuring 360 × 50 × 20 mm^3^ (L × T × R), was assessed in accordance with EN 1534:2000 [17]. A hardened steel ball with a diameter of 10 ± 0.01 mm was used to create five indentations in the tangential and radial surfaces of each specimen. The diameters of the measuring tool were measured with an accuracy of ±0.2 mm both along and across the grain after the indenter was removed for at least three minutes. The Brinell hardness was calculated using the mean value.

## 3. Results

### 3.1. Physical Parameter Changes and Mass Loss

**Figure 1:**
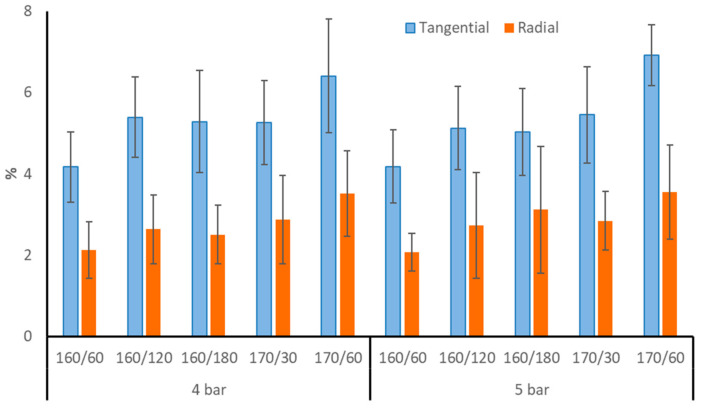
Aspen wood tangential and radial direction reduction after TM.

Following TM, the dimensions of aspen wood boards fell dramatically along both the tangential and radial axes. (Figure 1). The loss in TM aspen wood was higher in the tangential direction (4–7%) than in the radial direction (2–3.7%). After all treatments at 170 °C, the aspen wood’s tangential and radial directions showed the biggest modifications; nevertheless, there was no discernible difference between initial pressures of 4 and 5 bar. Silver birch and Scots pine wood likewise showed higher tangential reduction than radial after the identical TM process in nitrogen under pressure [4]. TM often induces shrinkage of wood after treatment in the tangential and radial directions, with minor longitudinal shrinkage. Wood with a greater ML after TM often has superior dimensional stability and biological durability, but its mechanical strength is diminished. ML is usually produced as a result of modifications to wood’s chemical composition. Most of the ML is caused by the breakdown of hemicelluloses, which are very susceptible to heat damage [5,18].

**Figure 2:**
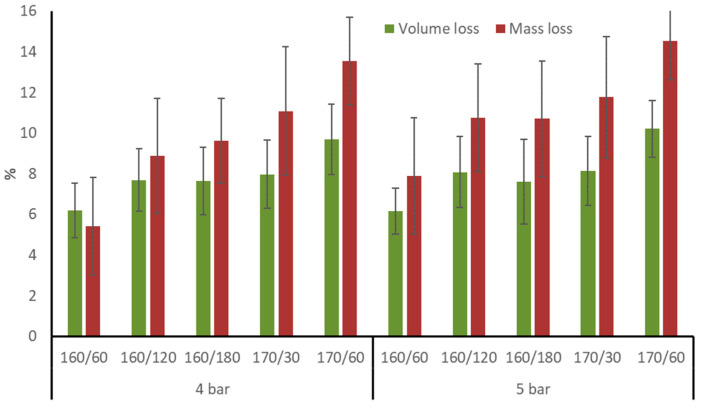
Aspen wood volume and mass loss after TM.

Following TM, aspen boards saw an average volume loss of 6.2 to 10.2% due to radial and tangential direction reduction (Figure 2). After the same TM treatment, birch wood showed similar average volumetric loss (7.1 to 10.2%), whereas pine wood was substantially lower (4.4–6.9%) [4]. Aspen wood’s average ML after TM varied between 5.4 and 14.5%. While initial pressure had no discernible impact, the average values of ML and volumetric loss rose with T_max_ and time at T_max_. After 1.5 to 3.5 h of TM at 185 °C in a nitrogen flow at 10 bar pressure, the ML of beech wood was 11–17% and that of pine wood was 8% [19]. Birch wood displayed lower resistance to TM in nitrogen because its greater average ML (5.9–12%) compared to pine wood (2.6–9%). Similar average ML was produced by TM in an open system in a nitrogen flow at T_max_ more than 200 °C and by TM in a closed system under pressure in nitrogen at 170–180 °C [4]. Aspen wood showed equivalent average ML after TM in nitrogen to birch, with the exception of regimes 170/60/4 and 170/60/5, which had higher average values.

### 3.2. Antiswelling Efficiency and Cell Wall Water Capacity

TM wood is mainly used for exterior cladding and decking, which corresponds to use class 3 according to EN 335. In such cyclical outdoor conditions, dimensional stability is very important. ASE shows the percentage difference between modified and untreated wood swelling. A higher value means that the particular treatment gives the wood better dimensional stability. To bring the test closer to real outdoor conditions, where the material is cyclically exposed to high humidity, it is important to find out how the dimensional stability changes during several soaking-drying cycles.

**Figure 3:**
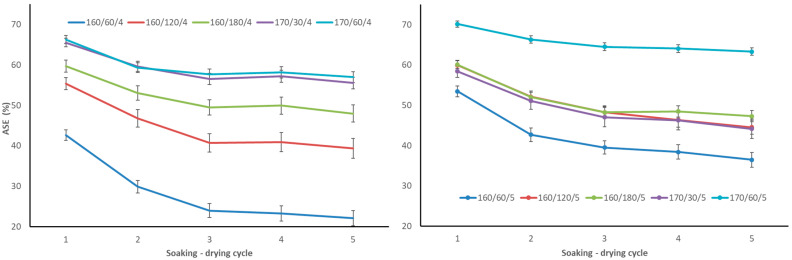
Antiswelling efficiency (ASE) of TM aspen wood during 5 soaking–drying cycles (**left**—TM regimes with initial pressure 4 bar, **right**—TM regimes with initial pressure 5 bar).

**Figure 4:**
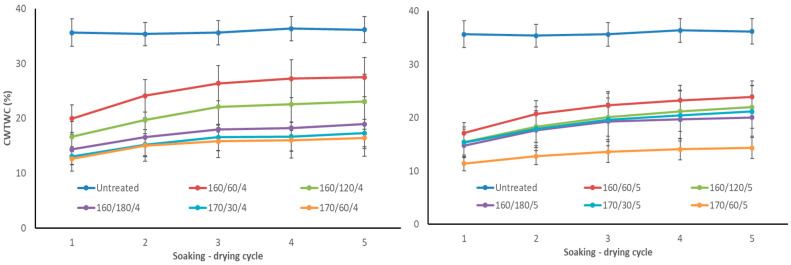
Cell wall total water capacity (CWTWC) of TM aspen wood during 5 soaking–drying cycles (**left**—TM regimes with initial pressure 4 bar, **right**—TM regimes with initial pressure 5 bar).

Following the first cycle, the ASE values of aspen wood after TM ranged from 42 to 70% (Figure 3). ASE decreased to 30–66% and 24–64%, respectively, following the second and third soaking-drying cycles. However, it remained relatively stable after the fourth and fifth cycles, reaching 22–63%. The reason for this decline is because TM wood’s structure becomes more water-accessible due to the leaching of thermal degradation products (Figure 4). The ASE values demonstrated a considerable difference between 4 and 5 bar starting pressures. At an initial pressure of 4 bar and a temperature of 160 °C, the ASE values increase noticeably by 22, 39, and 48% as the TM time rises from 60, 120, and 180 min. With ASE ranging from 56 to 57%, there was no discernible difference between the two treatments at 170 °C. At an initial pressure of 5 bar, samples TM at the regime 170/60/5 attained a much higher ASE value of 63% after the fifth cycle. The lowest ASE (36% after the fifth cycle) was achieved with the mildest TM regime 160/60/5, which is much higher than with 4 bar starting pressure. The remaining regimes, 160/120/5, 160/180/5, and 170/30/5, exhibited ASEs ranging from 44 to 47 percent and negligible error margins. Only regime 170/30/5 resulted in a substantial decline in ASE when compared to 170/30/4. All other samples after TM at 5 bar had higher ASE values at the same T_max_ and time at T_max_. This may have been influenced by the internal defects of the samples after TM. As shown by visual evaluation (see Table 1), the boards processed at 5 bar initial pressure had a higher percentage of defects than those processed at 4 bar. In the 170/60/5 regime, the percentage of defects was even more than half (55%), but this did not have a negative effect on the ASE of the samples. According to our previous research, regime 170/60/4 had the highest ASE (63% after the fifth cycle), and regime 170/30/6 had the lowest ASE for birch wood. Since TM produced the fewest defects and the highest ASE values, birch wood was better suited for an initial pressure of 4 bar rather than 6 bar [4]. Therefore, regimes with an initial pressure of 4 and 5 bar were also investigated in this study with aspen wood.

Maple (*Acer pseudoplatanus* L.) and ash (*Fraxinus excelsior* L.) wood were TM at 200 °C for 3 h, with slow (112 h) and quick (65 h) heating rates. Maple ASE was 47.9 and 38.6% at slow and quick heating rates, whereas ash ASE was 59.3 and 53.2%, respectively [20]. However, it is unclear how many soaking-drying cycles were undertaken. It is reasonable to believe there was only one cycle. As a result, it is unclear how these modifications will perform over time. Sapwood and heartwood from Scots pine (*Pinus sylvestris* L.) were TM in saturated steam at 120, 150, and 180 °C. The highest ASE values (60% for sapwood and 52% for heartwood) were achieved after TM at 180 °C, whereas the lowest for wood TM at 120 °C cycles [21]. Additionally, the authors do not provide the number of ASE cycles.

Prior research indicates that after TM in steam in a closed pressurised process at 160, 170, and 180 °C, the ASE for aspen (*Populus tremula*), grey alder (*Alnus incana*), and birch (*Betula pendula*) wood was 25–40%, 40–50%, and 55–65%, respectively, after the fifth cycle [22]. Aspen wood was able to achieve higher ASE after TM at T_max_ 160 and 170 °C under pressure in nitrogen, compared to treatment in a steam environment under pressure at the same T_max_. Our obtained ASE data after TM at 170 °C exceeded the ASE values after treatment in the steam environment under pressure at T_max_ 180 °C. Thus, TM under pressure in nitrogen is a more effective technique than TM in steam environments because the wood manages to get better dimensional stability by reducing the total processing cycle time from 24–30 h to 16–19 h.

TM aspen wood cell wall total water capacity (CWTWC) was reduced to 11–27% (Figure 4) compared to untreated aspen wood (35–36%). Over the course of five soaking-drying cycles, the CWTWC for every TM specimen grew somewhat. It is explained by the leaching of TM wood’s thermal degradation products. After the fourth cycle, there were slight CWTWC changes since most of the extractives were leached out in the first, second, and third cycles. CWTWC also demonstrated a substantial difference between 4 and 5 bar starting pressures. At an initial pressure of 4 bar and a temperature of 160 °C, CWTWC reduced marginally from 27 to 23% as TM time at T_max_ increased from 60 to 120 min. The remaining regimes, 160/180/4, 170/30/4, and 170/60/4, exhibited CWTWC values ranging from 17 to 19% and did not differ appreciably. At an initial pressure of 5 bar, the samples TM at regime 170/60/5 had the lowest CWTWC (14%) after the fifth cycle, as well as the highest ASE (Figure 3). At 5 bar initial pressure, the CWTWC for the remaining regimes varied between 20 and 24% and did not differ substantially. Regimes 160/180/5 and 170/30/5 had higher CWTWC at 5 bar than at 4 bar initial pressure.

Similar results were shown in our previous experiments. After TM in nitrogen, the CWTWC of birch wood decreased to 10–29% as opposed to 32–35% for untreated birch wood. In comparison to untreated pine wood (32–35%), TM pine wood’s CWTWC decreased to 14–27%. Over the course of five soaking-drying cycles, the CWTWC for every specimen increased somewhat [4]. Aspen (*Populus tremula*), birch (*Betula pendula*), and grey alder (*Alnus incana*) wood showed a similar propensity after TM at 160–180 °C in saturated steam in a closed system under pressure, according to a previous study [22].

### 3.3. Equilibrium Moisture Content

Because it affects mechanical strength, dimensional stability, and biological resistance, EMC content is an important characteristic of wood. Wood with a low EMC content absorbs less moisture and is less likely to swell and shrink in response to changes in the surrounding relative humidity (RH).

**Figure 5:**
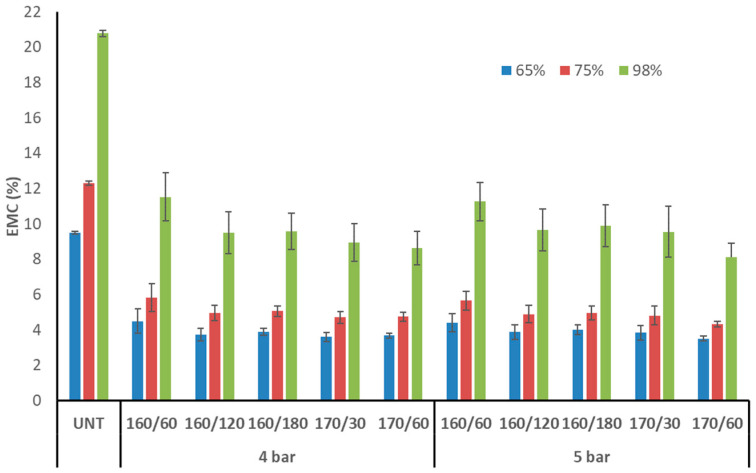
Equilibrium moisture content (EMC) of TM aspen wood at different relative humidity (UNT–untreated aspen wood).

At 65, 75, and 98% relative humidity, the EMC of untreated aspen wood was 9.5, 12.3, and 20.8%, respectively (Figure 5). The EMC of TM aspen wood dropped to 3.5–4.5%, 4.3–5.8%, and 8.1–11.5% at 65, 75, and 98% relative humidity, respectively. The EMC of TM aspen wood was 44–65% lower than that of untreated wood, with notable decreases in each RH. A previous study with silver birch and Scots pine after TM under nitrogen pressure found similar EMC reductions of 51–64% and 29–56%, respectively, at all RH levels [4]. When the T_max_, time at T_max,_ and initial pressure were increased, the average EMC values decreased. However, the adjustments were not important since the error bounds overlapped. Our earlier research demonstrated that ASE is a better method than EMC for detecting differences between distinct TM regimes in TM birch and pine wood [4]. The current investigation confirmed that the selected wood species does not matter, as the EMC after TM lowers to the same extent and there is no important difference between the treatment regimes.

The EMC at 76% of RH of poplar (*Populus nigra* L.) wood after TM at 160, 190, and 220 °C in superheated steam for 2 h was reduced to 10.5, 8.6, and 6.2%, respectively, compared to untreated wood EMC 10.6%. EMC reduction after TM was 19–41% [23]. In the steam environment, even at a greater T_max_, the EMC improvement was smaller than in our study following TM in nitrogen at pressure and lower temperature. Poplar wood density (384 kg × m^−3^) is closer to aspen wood (498 kg × m^−3^) compared to other hardwood species commonly used for TM (beech, birch, ash, oak, maple). It appears that less dense poplar microstructure after TM is more susceptible to moisture from the air than aspen wood. The EMC of beech wood (*Fagus sylvatica*) was 5–7% (65% of RH) and 13–23% (100% of RH) following two hours of TM in nitrogen at 175 °C and three hours of TM at 185 °C at 10 bar of pressure, respectively. In contrast, the EMC of untreated wood was 10% and 30%. When heated to 185 °C, beech wood TM had the lowest EMC among the specimens [24]. According to the methodology employed in this and a prior study [4], TM in nitrogen produced noticeably lower EMC values than those seen in the literature. The EMC of TM aspen at all RHs tended to decrease if T_max_, duration at T_max_, and initial pressure were promoted.

**Figure 6:**
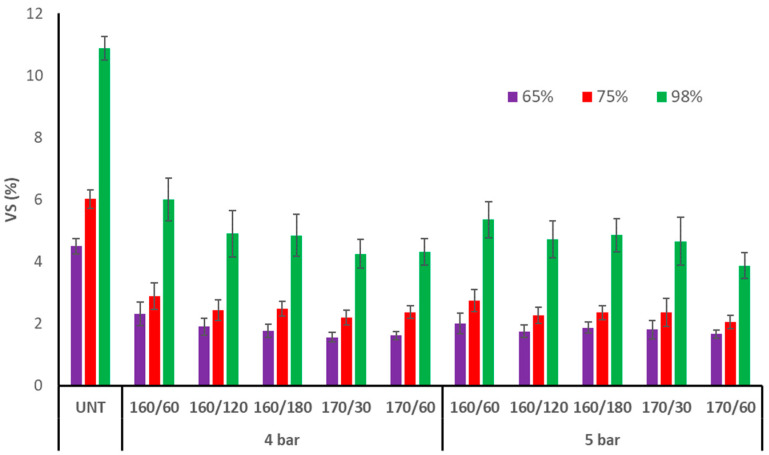
Volumetric swelling (VS) of TM aspen wood at different relative humidities (UNT–untreated aspen wood).

At 65, 75, and 98% relative humidity, the VS of untreated aspen wood was 4.5, 6.0, and 10.9%, respectively (Figure 6). The VS of TM aspen wood dropped to 1.6–2.3%, 2.0–2.9%, and 3.9–6.0% at 65, 75, and 98% RH, respectively. TM of aspen wood resulted in 45–67% reduced VS than untreated wood at all RH values. A previous study with silver birch and Scots pine found similar VS reductions of 48–67% and 29–61%, respectively, across all RH levels [4]. VS average values dropped as T_max_, time at T_max_, and initial pressure rose; however, because the error limits overlapped, the differences were important.

### 3.4. Bending Strength

**Figure 7:**
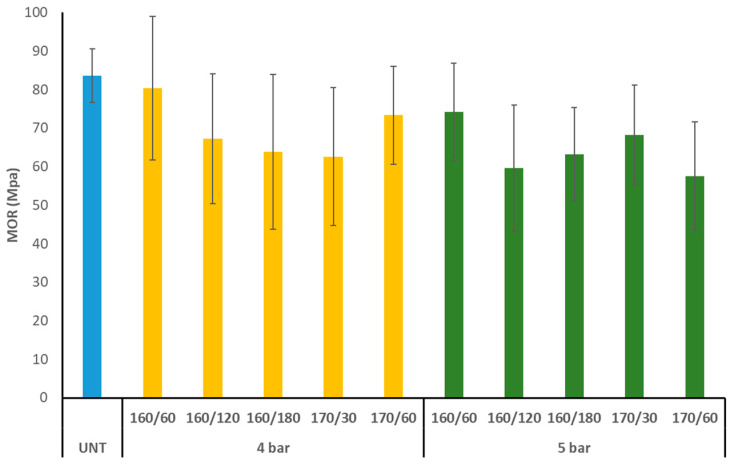
Modulus of rupture (MOR) of TM aspen wood (UNT–untreated aspen wood).

The MOR of untreated aspen was 84 MPa, which was lowered by 5–31% for all TM specimens (Figure 7). The lowest MOR values were obtained for regimes 170/30/4, 160/120/5, and 170/60/5 (58–63 MPa). TM of the samples resulted in a larger range of MOR values compared to untreated wood. Within the error limits, there were no appreciable differences between the initial pressures of 4 and 5 bars or between the TM regimes.

The MOR of silver birch (*Betula pendula*) and Scots pine (*Pinus sylvestris*) wood species decreased after TM under nitrogen pressure. For all TM specimens, the MOR was reduced by 15–42% from the untreated birch’s MOR of 124 MPa. In comparison to birch wood, untreated Scots pine had a MOR of 98 MPa and experienced a reduced overall MOR loss (2–32%) following TM. Changes in chemical composition following TM, particularly xylan degradation and acetyl group breakage, resulted in a decrease in the MOR. Hardwood (silver birch) was less thermally stable than softwood (Scots pine), as seen by reduced ML, extractive content, and the loss of xylan and acetyl groups [5]. Whereas birch wood’s TM in nitrogen under pressure produced the largest MOR deposition, aspen wood’s MOR reduction is comparable to that of pine wood.

**Figure 8:**
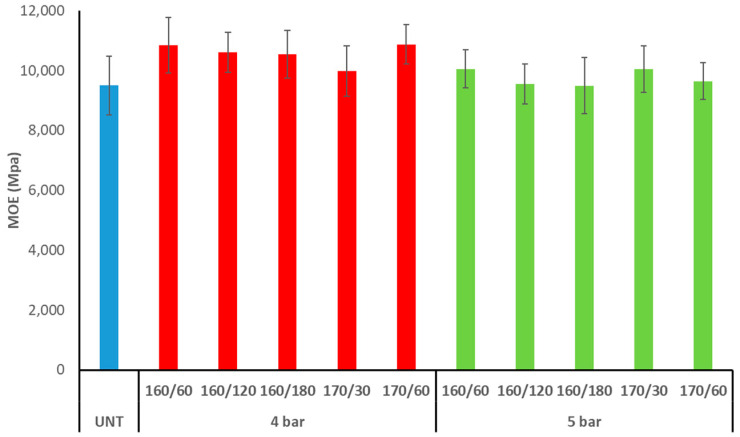
Modulus of elasticity (MOE) of TM aspen wood (UNT–untreated aspen wood).

The untreated aspen MOE was 9500 MPa, and treatment increased the average values depending on the treatment settings (Figure 8). The only regimes that yielded results equivalent to the untreated control were 160/120/5 and 160/180/5. It was discovered that specimens exposed to TM at 160/60/4 and 170/60/4 had the highest MOE (10,800 MPa). Nonetheless, there were few variations across all TM regimes, and the MOE values had large error margins. Similarly, there were no discernible differences in regimes with initial pressures of 4 or 5 bar. Additionally, following TM under nitrogen pressure, the MOE changes of Scots pine (*Pinus sylvestris*) and silver birch (*Betula pendula*) wood species were negligible [5].

MOR of maple (*Acer pseudoplatanus* L.) and ash (*Fraxinus excelsior* L.) wood following TM at 200 °C for 3 h using slow (112 h) and fast (65 h) heating rates was reduced from 128.8/131.8 Mpa for untreated wood to 99.6/83.8 Mpa and 82.6/91.0 Mpa after slow/fast heating rates, respectively. MOE was less affected by TM than MOR. Only fast-modified maple wood showed a considerable reduction in MOE (35%) [20]. Thermowood treatment at 180 °C lowered the MOR of European (*Populus tremula* L.) and hybrid (*P. tremula × tremuloides*) aspen wood from 71.5/68.0 Mpa to 65.4/58.7 Mpa. MOE values after TM increased to 14.2/12.3 Gpa compared to the untreated European and hybrid aspen MOE 12.6/12.0 Gpa, respectively [11]. After TM in the oven between metallic plates to 230 °C in a closed process under nitrogen or vacuum (200 mbar), beech (*Fagus sylvatica*) wood’s MOR dropped by roughly 45% and 25%, MOE by 12% and 8%, and Brinell hardness by 22% and 2%, respectively. These findings support prior research demonstrating that samples handled under vacuum degrade less than those treated with nitrogen [10].

### 3.5. Brinell Hardness

**Figure 9:**
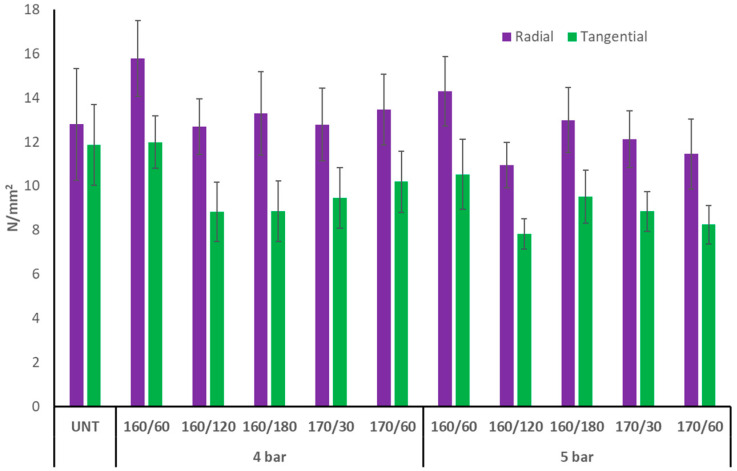
Brinell hardness of TM aspen wood radial and tangential surfaces (UNT–untreated aspen wood).

Brinell hardness (BH) was considerably higher on the radial surface of TM aspen wood than on the tangential surface (Figure 9). The untreated aspen wood BH of both anatomical surfaces was similar (12 to 13 N × mm^−2^). After each treatment, the BH of the TM aspen wood tangential surface decreased. The radial surface BH of TM aspen wood increased, especially under the mildest treatment regimens of 160/60/4 and 160/60/5. Perhaps the tangential shrinkage (see Figure 1) of aspen wood at 160 °C compacted the radial surface, but thermal degradation of chemical components and ML was lower than in 170 °C treatments, resulting in a minor increase in BH. Between the TM regimes with initial pressures of 4 or 5 bar, there was no discernible difference in BH values.

The tangential surface of TM birch wood had a BH far greater than the radial surface, but Scots pine wood displayed the reverse trend. This is because the two examined wood species exhibit different microstructural rearrangements following TM in nitrogen [5]. After TM, aspen wood demonstrated a similar trend in MOR and BH alterations for various anatomical surfaces as pine wood.

Sapwood and heartwood of Scots pine (*Pinus sylvestris* L.) were treated with saturated steam at 120, 150, and 180 °C in a high-pressure reactor. Wood TM at 180 °C decreased sapwood/heartwood BH to 9.4/9.9 N × mm^−2^, compared to untreated wood’s BH of 11.6/11.4 N × mm^−2^ [21]. Scots pine wood (*Pinus sylvestris* L.) was TM at 200 °C, atmospheric pressure, and air for 4, 6, and 8 h. The MOR of untreated pine was 81 Mpa, which dropped to 81, 70, and 60 Mpa after 4, 6, and 8 h of TM, respectively. MOE fell marginally to 9100, 9200, and 9000 Mpa after TM, compared to the untreated wood MOE of 9500 Mpa [25].

After TM at 200 °C for 3 h, the BH of maple (*Acer pseudoplatanus* L.) wood decreased both tangentially and radially due to rapid heating (65 h). There were no appreciable differences between the other groups, with the exception of the BH in the tangential direction for slow (112 h) modified ash (*Fraxinus excelsior* L.) wood. The authors claim that the more porous structure of ash wood and the distinctions between early and late wood sometimes affected the tangential measurements, reducing the accuracy of the BH results. Depending on the species, kind of TM used, test procedure, and test instructions, the hardness values reported for TM wood are usually inconsistent, showing both greater and lower results when compared to untreated wood in the literature [20]. Black poplar (*Populus nigra* L.) was treated with superheated steam at 160, 190, and 220 °C for 2 h. Higher treatment temperatures dramatically reduced the MOR and BH. These connections were especially evident at higher temperatures (190 and 220 °C), when TM wood exhibited increased hemicellulose breakdown. The MOR of untreated poplar in the absolute dry condition was 70.0 Mpa, which decreased to 55 and 30 Mpa at 190 and 220 °C, respectively. The MOE after TM was minimally influenced [23]. After TM at 180 °C using the Thermowood technique, the European aspen (*Populus tremula* L.) BH increased from 15.0 Mpa for untreated wood to 15.8 Mpa for TM wood. After the same TM, hybrid aspen (*P. tremula × tremuloides*) showed a decrease in BH from 14.2 Mpa for untreated wood to 13.3 Mpa for TM wood. However, the average densities of both species employed for TM were lower (~420 kg × m^−3^) than aspen in our investigation (average density 498 kg × m^−3^) [11].

## 4. Discussion

In the scientific literature, ASE is a widely used approach for characterising wood and its products after various alterations. This metric compares the performance of the modification process to the raw material. Since ASE makes it possible to compare the effectiveness of various methods, it is one of the procedures that needs to be tested for modified materials. However, studies rarely state how many ASE soaking-drying cycles were performed. If not indicated, it is assumed that only one cycle was performed. Our comprehensive research with numerous materials and modification approaches reveals that ASE values after the first cycle do not accurately reflect the material’s long-term dimensional stability. In the second and third cycles, ASE values decrease by 5–10% in absolute terms and 2–5%, respectively. There are only little variations in the fourth and fifth cycles, but no appreciable shifts in values. The decrease in ASE shows that thermal degradation chemicals are leaching out of TM wood, making its microstructure more susceptible to water absorption, which is followed by cell wall swelling. The decline in ASE during the second and third cycles of impregnation alterations suggests that the wood structure’s poorly bound impregnation chemicals are leaching out, and that the microstructure is more vulnerable to water action. As a result, the material available in the scientific literature should be thoroughly analysed, and the huge improvement in features that is commonly mentioned is only visible, but it is much smaller or does not exist at all.

Surprisingly, changes in the mechanical strength of aspen wood followed a similar pattern as previously examined pine wood but not birch wood. Clearly, the microstructure of untreated aspen wood is more comparable to birch and distinct from pine wood. Overall, there were no appreciable differences in MOR, MOE, or BH between aspen TM regimes. Boards having a density of 498 ± 30 kg × m^−3^ were selected for TM. Despite careful sample selection, mechanical strength fluctuated dramatically following processing. When compared to untreated wood, only aspen BH demonstrated a discernible reduction in tangential surface. Following the TM, the ML of the aspen boards was in a wide range; as a result, their density is not uniform, and mechanical strength indicators are higher than those of the raw material. Solid wood is highly heterogeneous; the board’s volume may be comprised of both heartwood and sapwood. The average density can vary greatly depending on the region, even if it is the same for all boards. By cutting the board into smaller pieces, samples with a variety of densities and features can be obtained. Chemical changes determine the qualities of wood following thermal treatment. When doing chemical studies on wood, an average sample is frequently obtained from a variety of boards at different areas. Following TM, the chemical structure of a solid wood board is likely to change dramatically. This can be altered by the sapwood/heartwood ratio, the board’s position (ends or centre), and the board’s location in the modification chamber. A separate investigation should be conducted to confirm this hypothesis.

Aspen appears to be a potential material for TM. It is relatively inexpensive, and after TM, it develops a dark brown colour that imitates much more expensive material. Our investigation provided useful information concerning evident faults (cracks, bloating, twisting, etc.) that helped us describe the general condition of the boards after processing. This is not common in literature. In our experience, 10–20% of boards fail after TM, which is both frequent and promising.

## 5. Conclusions

Aspen wood’s structural stability was enhanced, and moisture and water absorption were decreased by TM in a closed process under nitrogen pressure. TM caused 6.2–10.2% ML and 7.1–10.2% volume loss. Following TM, aspen wood’s ASE values ranged from 22 to 70%. Additionally, it tended to decrease throughout the course of five soaking-drying cycles due to the leaching of thermal breakdown products, which increased the CWTWC and increased the susceptibility of the wood structure to moisture. TM under pressure in nitrogen was approved as a more effective technology than TM in a pressurised steam environment because the wood achieved higher dimensional stability while lowering the overall processing cycle time from 24–30 h to 16–19 h.

TM aspen wood had 44–65% lower EMC and 45–67% lower VS than untreated wood. The MOR was lowered by 5–31 percent for all TM specimens. The MOE values increased following the majority of treatments, however, the variations across all TM regimes were negligible. The BH of the TM aspen wood’s tangential surface dropped with each treatment, but the radial surface BH increased after a few treatments or remained equivalent to untreated wood.

With initial pressures of 4 or 5 bar, the majority of the procedures under study did not demonstrate any discernible differences between the TM regimes. Only ASE results showed important differences between tested pressures. Despite the fact that specimens treated under regimes with 5 bar initial pressure demonstrated greater dimensional stability, 4 bar is more appropriate for TM of aspen wood. This recommendation was clearly approved by the material quality inspection following TM (see Table 1). TM at 5 bar pressure caused more dramatic material breakdown, resulting in a greater percentage of boards with evident faults.

## Figures and Tables

**Table 1:** Thermal modification parameters of European aspen wood.

Treatment Regime	T_max_ (°C)	Time at T_max_ (min)	Initial Pressure (bar)	Max Pressure (bar)	Boards with Defects After TM
160/60/4	160	60	4	11.0	0 (0%)
160/120/4	160	120	4	11.7	0 (0%)
160/180/4	160	180	4	11.9	0 (0%)
170/30/4	170	30	4	13.7	2 (11%)
170/60/4	170	60	4	15.0	4 (22%)
160/60/5	160	60	5	12.8	1 (5%)
160/120/5	160	120	5	13.3	2 (11%)
160/180/5	160	180	5	13.9	4 (22%)
170/30/5	170	30	5	15.0	4 (22%)
170/60/5	170	60	5	17.4	10 (55%)

## Data Availability

The original contributions presented in this study are included in the article material. Further inquiries can be directed to the corresponding author.
